# Impact of Element Size on Rebar–Concrete Interface Microstructure Using X-ray Computed Tomography

**DOI:** 10.3390/ma17153873

**Published:** 2024-08-05

**Authors:** Milena Kucharska, Piotr Dybeł

**Affiliations:** Faculty of Civil Engineering and Resource Management, AGH University of Krakow, Al. A. Mickiewicza 30, 30-059 Krakow, Poland; dybel@agh.edu.pl

**Keywords:** X-ray computed tomography, self-compacting concrete, steel–concrete interface, air voids, settlement

## Abstract

This paper investigates the impact of element size on the microstructure of the steel–concrete interface in self-compacting concrete (SCC). Experiments were conducted on two types of test elements: a deep beam measuring 1440 × 640 × 160 mm and a wall element measuring 2240 × 1600 × 160 mm. The SCC mix was consistently pumped from the top, using a single casting point located near the formwork’s edge. Horizontal steel ribbed rebars with a diameter of 16 mm were embedded in these elements. X-ray computed tomography (CT) was employed to provide three-dimensional insights into the microstructure of the rebar-to-concrete interface. An analysis of X-ray CT images from core samples revealed that the microstructure of this interface is influenced by the distance of the specimen from the mix casting point and its vertical position within the element. The combined effects of bleeding, air-pore entrapment, and plastic settlement within the SCI were observed under the top rebars. Their extent was independent of the type of element analyzed, suggesting that the deterioration of the SCI is related to the distance from the top surface of the element. These results elucidate phenomena occurring during the fresh state of concrete near reinforcing bars and their implications for bond properties. To date, some of the standards differentiate between bond conditions according to the depth of concrete beneath the rebar. In the view of the studies, this approach may be unduly rigorous. The findings offer valuable guidance for reinforced concrete execution and design.

## 1. Introduction

The structural performance of reinforced concrete elements heavily relies on the interaction between the reinforcement steel and the surrounding concrete, commonly known as the steel-to-concrete bond. The ultimate homogeneity of the structural material primarily hinges on the effective bonding of concrete to the reinforcement steel, making it one of concrete’s most crucial hardened properties. Bond strength is widely acknowledged to be significantly influenced by the properties of hardened concrete and reinforcement, as well as the compaction of fresh concrete before hardening. Inattentive or overly vigorous formwork filling can result in areas with potentially more favorable compaction conditions at the bottom compared to those at the top. This is related to phenomena during the consolidation process of fresh concrete, such as bleeding and plastic settlement—where water migrates from the bottom to the top surface, and the concrete settles downwards, respectively.

The structural behavior and durability of reinforced concrete are significantly influenced by the steel–concrete interface (SCI), making it a fundamental aspect in the engineering of such structures. The quality of the SCI is directly reflected in the steel–concrete bond, affecting it in terms of adhesion and mechanical interlocking between the steel and concrete [1]. Negative phenomena within the SCI, such as segregation, settlement, and bleeding of fresh concrete, can lead to the formation of air voids under horizontal rebars [2]. Additionally, air entrapment under horizontal rebar surfaces may occur due to deaeration of the mix, where rising air bubbles adhere to the rebar. These air voids can vary considerably in size and shape. For instance, voids formed by bleed water tend to be elongated or crescent-shaped, potentially achieving a larger contact zone with the rebar surface in comparison to other air voids. However, since bleed-water voids initially hold water and can later be emptied due to chemical shrinkage and absorption through cement-paste hydration or evaporation, they can be mistakenly identified as entrapped air voids. Such voids can have significant implications for corrosion susceptibility. The mentioned phenomena within the steel–concrete interface (SCI) significantly degrade the quality of the steel–concrete bond, a factor directly impacting the practical implementation of standard design codes [3,4]. This effect has been considered in regard to international standards, where the reduction in bond strength is compensated for by appropriately extending the anchorage length.

It is observed that, in comparison to traditional concretes, new-generation concretes demonstrate a reduced propensity for the segregation of components or the retention of air. One exemplar of a new generation concrete is self-compacting concrete (SCC). It offers distinctive rheological properties that enable the complete filling of a mold or formwork of any shape without segregation or the need for additional mechanical compaction [5]. A secondary advantage, which is also a requirement for a self-compacting mix, is its ability to flow through densely packed reinforcement. The high fluidity of the SCC mix ensures not only the flow and the self-levelling of its surface but also facilitates the natural release of air pores, following the principle of hydrostatic buoyancy. In particular, instances of negligence may result in the unintentional entrapment of air during the production or transportation process. Thus, the advantages of self-compacting concretes only limit the negative phenomena occurring in the interfacial transition zone between steel and concrete compared to normal concretes.

Given the relationship between settlement extent and element depth, negative phenomena within the SCI are expected to intensify on the bottom side of the rebars positioned closer to the top surface of the concrete element [6]. However, differences in SCI quality between the bottom and top sides of the rebar become apparent at greater distances from the bottom of the form, typically over 150 mm of concrete depth under the rebar [7]. The evaluation of SCI quality beneath the bottom surface of the rebar can be conducted using surface image analysis after splitting a sample following a pull-out test [8], employing video microscope analysis [9], or utilizing X-ray computed tomography (X-ray CT) [10]. Tests performed on normally vibrated concretes (NVCs) and self-compacting concretes (SCCs) of various compressive strengths (25 and 40 MPa) in 1.1 m high elements [9] revealed the presence of a bleed-water zone ranging from 0.1 to 0.2 mm for SCCs, irrespective of compressive strength. In NVCs, especially those with lower compressive strengths, wider voids were observed, up to 0.7 mm. More recent studies indicated that plastic settlement in fresh NVC could result in continuous voids forming beneath the rebar, extending up to 1 mm [11]. Moreover, the air voids’ thickness was affected by the distance of the rebar from both the casting surface (casting position factor) and the nearest vertical disruptions beneath the reinforcement. Based on findings for SCCs [10], the average settlement value of the concrete mix under the rebar was 0.55 mm. The size of air voids varied along the rebar’s length, with larger voids observed directly under the ribs (ranging from 0.48 to 1.18 mm), while voids between ribs measured up to 0.4 mm. The research also discussed the technology of SCC placing, highlighting the potential benefits of the bottom-up placing method. This method reduces the amount of air voids at the rebar–concrete interface and eliminates mixture settlement beneath the top rebar.

X-ray CT is a non-destructive imaging technique that is widely used in various scientific and industrial fields. It involves the acquisition of X-ray images of an object from multiple angles around its axis. The X-ray projections are subsequently processed to reconstruct detailed cross-sectional views of the object. X-ray CT provides valuable information about the internal structure, composition, and density distribution of materials with high spatial resolution across various fields of study [12]. Depending on the equipment and configuration used, the resolution usually varies from a few micrometers to a few millimeters per voxel. The most important limitations of this method are the size of the specimen, which must be adapted to fit the CT scanner, and the susceptibility to artifacts (e.g., beam hardening artifacts). Artifacts are distortions caused by dense materials, vibrations, or movement during scanning. However, in most cases, these artifacts can be minimized or eliminated during the reconstruction process. In civil engineering, it is particularly useful for detecting cracks, voids, and other defects within structural components, thus facilitating the assessment of their integrity and durability. It is mostly adapted in the evaluation of cementitious materials [13], such as cement paste [14], plain concrete [15], lightweight concrete [16], foamed concretes [17], or fiber-reinforced concretes [18]. Studies on the effects of workability and compaction time on the microstructure and pore distribution of concrete material have been conducted [19]. These variables have a significant effect on the distribution, shape and size of the pores, which contribute to the final durability and strength of the concrete. Therefore, finding the proper equilibrium between appropriate workability and sufficient vibration time is crucial to minimize the possibility of segregation. Moreover, the impact of surface treatment on the concrete skin was evaluated using X-ray CT [20]. It was demonstrated that different surface treatment methods result in varying thicknesses of the concrete skin. The influence of the air-entraining agents (AEA) was also determined using X-ray CT, which concluded that the addition of these agents increases the number of pores while decreasing their diameters and spacing [21]. In the field of reinforced concrete, research involving X-ray CT has focused various aspects, including the influence of corrosion pit distribution [22], the casting position factor [11], and the SCC placing method [10] on pore characteristics in the vicinity of rebar. Significant variations in air voids characteristics with increased pit dispersion around rebars was shown with an increase in both fine and large pores that facilitated crack formations [22]. X-ray CT has also been utilized in evaluating different civil engineering materials. The technique has been adapted to assess damaged structural elements from an old timber bridge [23] and to determine the extent of wood destruction after sclerometric testing [24]. X-ray CT has also been applied to detect discontinuities and porosity in stainless steel [25] and to evaluate the morphology and pore characteristics in silica bricks [26].

The general conclusion on the microstructure of the concrete–rebar interface is that its quality deteriorates with increasing depth below the rebar and decreasing concrete strength [9]. However, international standards [3,4] seem to be inconsistent when considering aspects dependent on the quality of SCI. In the current European standard EN 1992 [4], bond quality depends on the bar’s inclination to the horizontal and its position relative to the top surface during concreting. According to the American standard ACI 318-19 [3], the casting-position factor depends on the thickness of fresh concrete beneath the horizontal reinforcement. The employment of CT scanning technology facilitates the precise and detailed verification of the quality of the SCI in relation to numerous factors, including material and technological aspects. While previous research has extensively covered various aspects of SCC, including its composition and mechanical properties, the influence of element dimensions on the microstructural integrity of the rebar–concrete interface remains underexplored. This research provides a novel approach to understanding these effects, offering practical insights that can inform and improve construction practices and standards for SCC.

## 2. Materials and Methods

### 2.1. Materials

**Figure 1:**
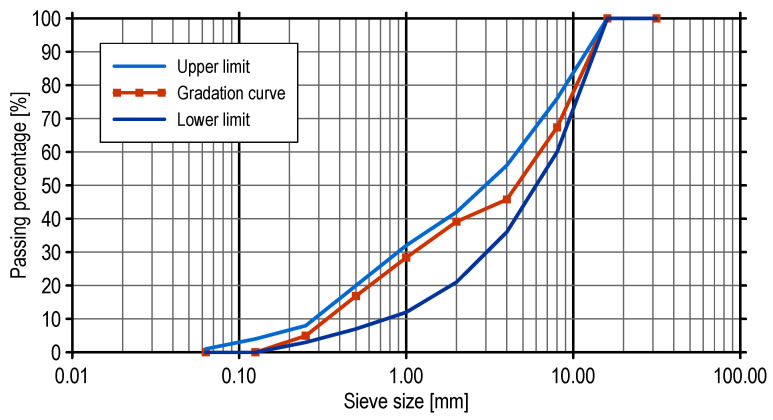
Gradation curve of the combined aggregates.

Table 1 outlines the composition of the SCC mixes chosen for further experimentation. The SCC mixture was developed by the ready-mix supplier. Blast-furnace cement (CEM III/A) with a strength class of 42.5 N/mm^2^ (ordinary early strength) was utilized in this study. The cement met the requirements of EN 197 [27]. Additionally, two gravel fractions (2–8 mm and 8–16 mm) were included as coarse aggregates, while a natural sand fraction of 0–2 mm served as the fine aggregate. The gradation curve of the combined fine and coarse aggregates is illustrated in Figure 1. The desired flow properties of the mixtures were achieved using varying amounts of a superplasticizer (polycarboxylic ether polymer) and plasticizer. The total binder content was maintained at 450 kg/m^3^, the minimum recommended value for SCC mixes. The water-to-binder ratio was fixed at 0.36.

Table 2 presents the results of both the flow properties of the fresh SCC mixtures and the compressive strength of the hardened SCC. The assessment of the flow properties allowed for classification into slump flow (SF1), viscosity (VS2), and passing ability (PL2) classes according to standard guidelines [28,29]. Additionally, the fresh visual stability index [30] was determined as 1, indicating the absence of segregation with a minor bleeding effect. The compressive strength of SCC was determined using 11 cubic samples, each with a side dimension of 150 mm.

The tests were conducted using ribbed reinforcing bars (B500SP). The rib pattern of B500SP rebar comprises two rows of transverse ribs and, typically, two longitudinal ribs. The transverse ribs on each side of the bar are alternately positioned at two different angles relative to the longitudinal axis. A diameter of 16 mm was used as the representative diameter within the range of mean diameters (10–20 mm) [31].

### 2.2. Specimens and Basic Modules

**Figure 2:**
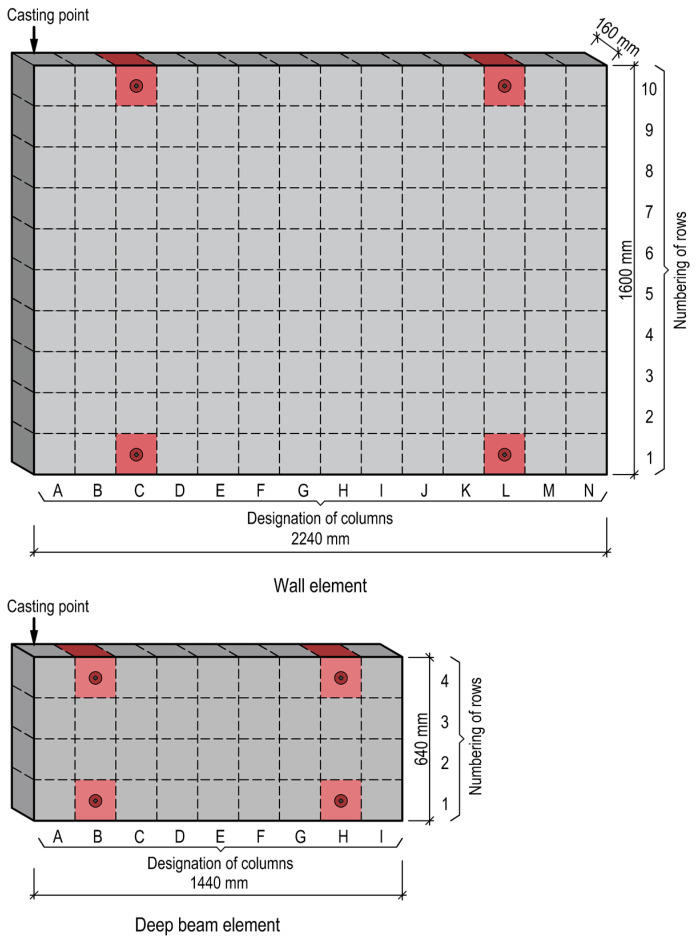
Schematic view of the wall and deep beam elements.

**Figure 3:**
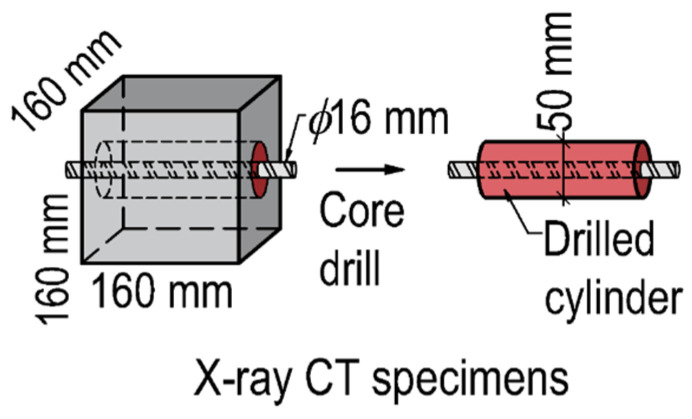
X-ray CT module sample.

Two types of elements of varying dimensions were manufactured for the research: a wall element measuring 2240 × 1600 × 160 mm and a deep beam element measuring 1440 × 640 × 160 mm. The specimens were designed to be divided into 160 mm cubes (Figure 2). From these elements, core specimens with centrally embedded reinforcing bars were extracted and prepared for SCI X-ray examination (Figure 3). The samples are marked in red in Figure 2. The remaining samples were subjected to further strength testing used in another study. Each element was marked with division lines corresponding to columns labelled with capital letters and rows numbered from 1 at the bottom. At one end of the element, in the area of column A, a single casting point was established.

Both the wall and beam elements were constructed using a pumped concrete mix. After 7 days of curing, the formwork was removed, and the specimens were subjected to a curing process in fixed positions under laboratory conditions with continuous water sprinkling. Subsequently, after 21 days of curing, the elements were cut into smaller cubic or core samples for testing. Due to the time-consuming nature of cutting and preparing the test elements, testing was conducted 40 and 68 days after concreting for the wall and beam elements, respectively. The timing of X-ray CT examinations does not influence the outcomes of the examinations.

### 2.3. Test Procedures

#### 2.3.1. X-ray Computed Tomography

**Figure 4:**
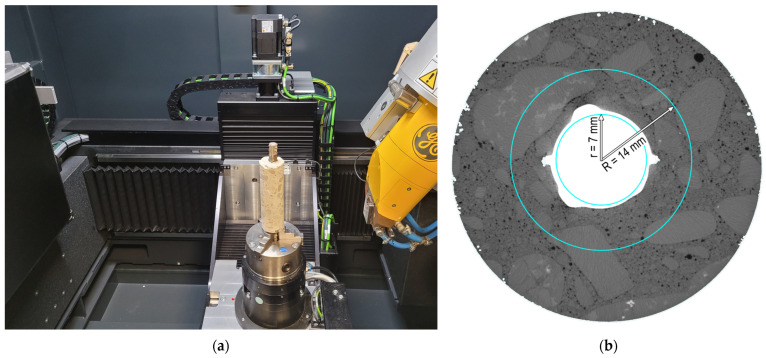
X-ray CT investigation details: (**a**) test setup and (**b**) region of interest for porosity analysis.

The core samples were examined using a GE Phoenix v|tomex|m X-ray CT system (Figure 4). During the X-ray CT investigation, the sample is positioned between the radiation source and the detector. The measurement adjustments are made by specifying the scanned object’s position relative to the source and detector and setting the voltage and current values for radiation generation. A 300 kV mini-focus source was employed for the test, emitting X-rays of suitable energy to penetrate the concrete element and reach the detector. By measuring X-ray beam attenuation, a detailed internal structure representation of the sample is created in the form of high-resolution 2D images at a 0.05 mm/pixel resolution. During scanning, the detector and source remain stationary, while the specimens rotate on a turntable along their vertical axis. All samples were scanned using consistent parameters, producing 1800 images per scan with an average duration of approximately 90 min.

#### 2.3.2. Volumetric Analysis

Following the X-ray CT scans, a reconstruction process was undertaken involving the integration of a series of 2D images into a 3D model using specialized software. The reconstruction process included beam-hardening correction, automatic geometry calibration, and geometry optimization algorithms. The resulting 3D model was then analyzed to detect and examine air voids around the rebar. A porosity analysis was conducted using Volume Graphics software: VGSTUDIO MAX 2024.2, within a defined Region of Interest (ROI) focused on the vicinity of the rebar. The ROI was specified as a cylinder around the rebar with an outer radius of 14 mm, an inner radius of 7 mm, and a length of 75 mm (Figure 4). These dimensions were carefully chosen to ensure adequate volume for an air-void analysis while optimizing the calculation time. To quantify sample porosity, the VGDefX algorithm was utilized in ‘Only threshold’ mode. Air voids, including pores and voids formed during mixture settlement, were visualized in distinct colors in both 2D and 3D views corresponding to their volume.

## 3. Results and Discussion

**Figure 5:**
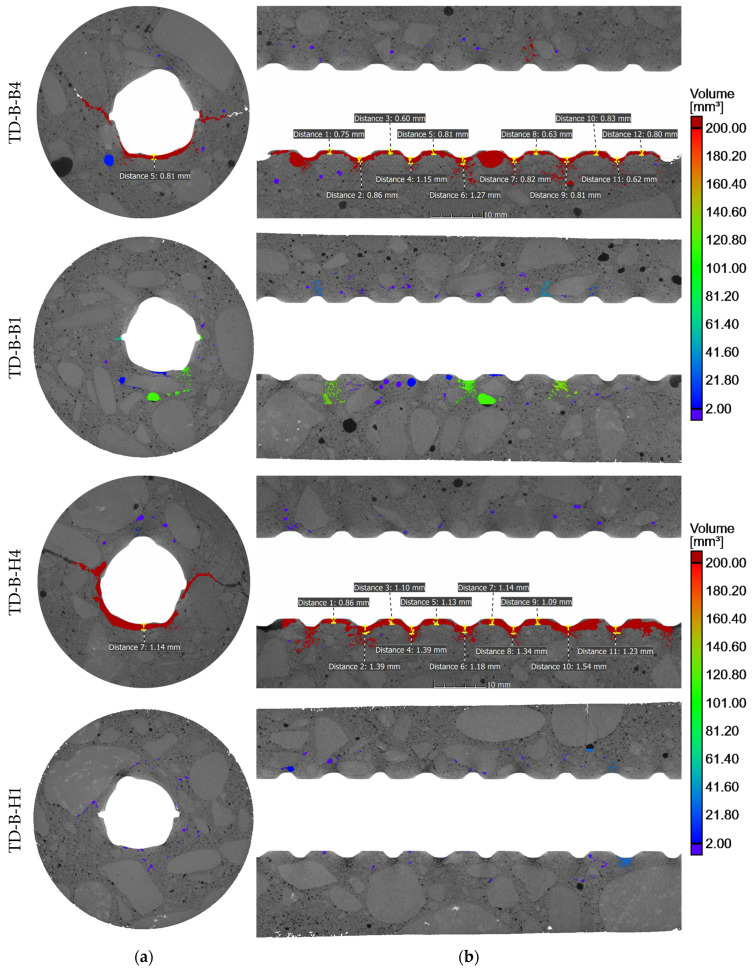
Tomography sectioning of concrete cores extracted from the deep beam element: (**a**) cross-section and (**b**) longitudinal section.

**Figure 6:**
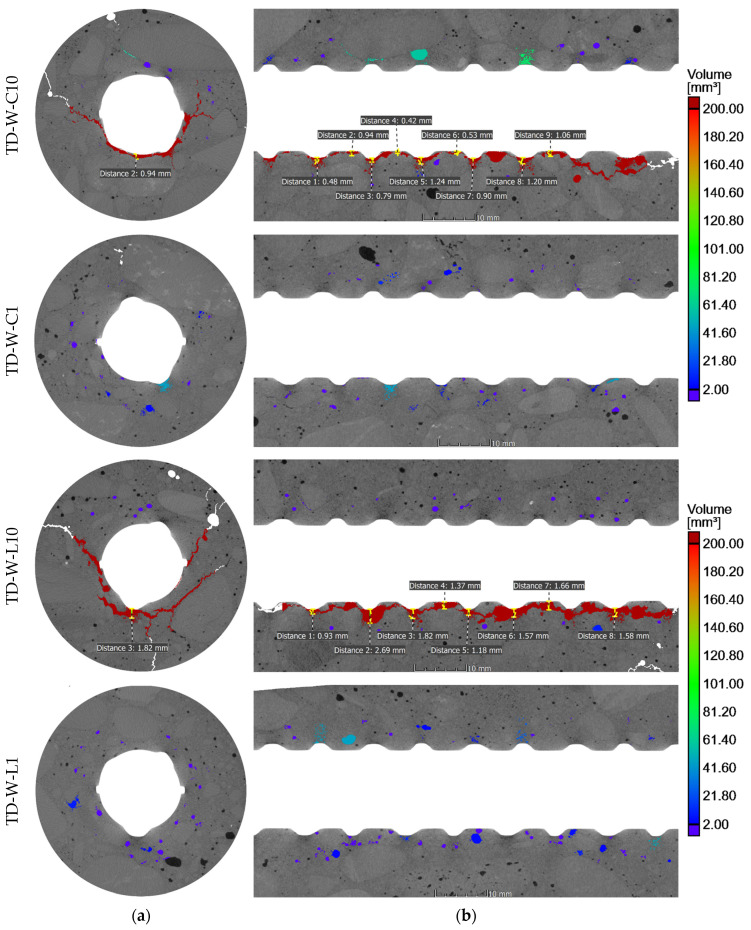
Examples of tomography sectioning of concrete cores extracted from the wall element: (**a**) cross-section and (**b**) longitudinal section.

**Figure 7:**
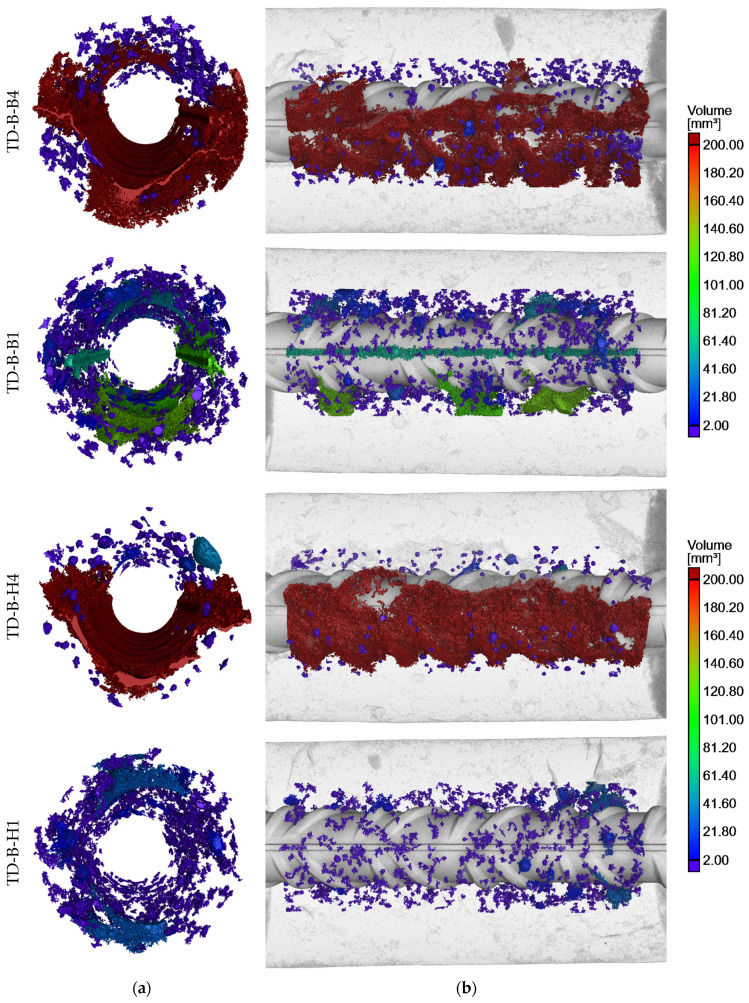
Reconstruction of porosity distribution around reinforcing bar in deep beam element: (**a**) 3D cross-section and (**b**) 3D longitudinal section.

**Figure 8:**
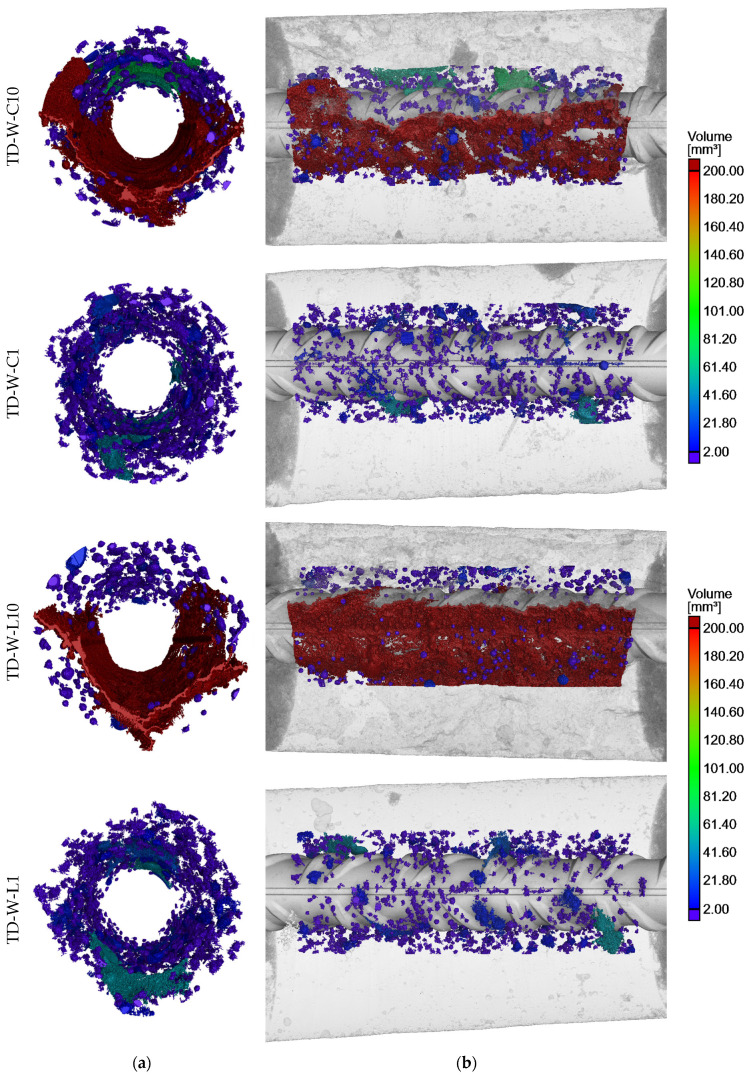
Reconstruction of porosity distribution around reinforcing bar in wall element: (**a**) 3D cross-section and (**b**) 3D longitudinal section.

The SCC core samples taken from the test elements underwent X-ray CT examinations, offering precise assessments of porosity and the visual presence of voids within concrete specimens. These examinations focused on areas near the rebars to detect voids caused by bleeding and settlement of the concrete. Those phenomena are commonly associated with air occurrence in fresh concrete in the form of bubbles and voids, resulting from intentionally entrained or unintentionally trapped air. The results of the porosity analysis around the rebars are summarized in Table 3. The key findings of the tomography imaging are depicted in Figure 5, Figure 6, Figure 7 and Figure 8, showcasing core samples collected at different depths (bottom and top samples), various distances from the casting point (near and further) from two types of test elements (a deep beam and a wall element) in both 2D and 3D views. The porosity analysis around the rebars specifically targeted bubbles and air voids exceeding 0.2 mm^3^ in volume, considered significant for this analysis. The color scale is fixed for all samples.

In the present study, porosity is considered to be the ratio of the volume of voids with a dimension smaller than 0.2 mm^3^ to the volume of the entire ROI. The porosity analysis revealed that the highest percentage of air in the steel–concrete interface zones was found in the samples located at the top parts of the element, regardless of its type. The porosity rate ranged from 5.68% to 7.46% for the top samples and from 1.34% to 3.37% for the bottom ones. Despite the fact that all of the top specimens achieved a relatively substantial and similar void volume around the rebar, the highest volume of air voids was observed in the H4 sample, which was located further from the casting point of the deep beam element.

X-ray CT examinations provided insight into the occurrence of both continuous voids under the entire length of rebar and entrapped air bubbles in the SCI. The approximate depth of settlement along the given length of rebars and air bubbles in the SCI is shown in Figure 5 and Figure 6. The measurements given in Figure 5 and Figure 6 were made only for air voids caused by the combined bleeding and plastic settlement effects. The voids caused by settlement were observed under the top rebars. The magnitude of those voids ranged from 0.6 mm to 1.54 mm for the deep beam element and from 0.42 mm to 2.69 mm for the wall element.

Large air pores trapped beneath aggregate grains were also noted in the top samples of both the deep beam and wall elements. The rest of the air pores in the SCI were predominantly smaller than 10 mm^3^, with a majority of those being below 2 mm^3^.

### 3.1. Effect of Rebar Position Over Height

In general, the negative phenomena around the rebars were more noticeable in the X-ray CT scans of top samples than the bottom ones. This trend is commonly known in the existing literature concerning SCI and bond phenomena [7,9]. The air voids that spread along the bottom surface of the top rebars were the result of the bleeding and consolidation of fresh concrete. Notably, the settlement of fresh concrete under the rebars was generally not uniform. The largest settlement depths primarily formed under the rib lugs, while smaller voids developed in the spaces between them (Figure 5 and Figure 6). It appears that the shape of the ribs facilitated the slipping of the mixture, thereby creating larger voids under the rebar ribs. The isolated air pores trapped between the ribs were exceptions to that trend. It is important to note that research on normal and self-compacting concretes [9] revealed that the bond between ribbed rebars and concrete is most adversely affected by the width of the void in the inter-rib area, which, in this case, is mostly smaller. The presence of voids between the ribs results in a reduction in the contact area between the rebar ribs and the concrete, or in the exclusion of individual ribs from the transfer of the pullout force. Consequently, in such a joint, a non-uniformity of the bond stress distribution along the rebar axis will occur, resulting in a reduction of the bond stiffness.

The average depth of concrete settlement below the rib lugs in sample B4 (deep beam) was 0.93 mm and 0.73 mm between the ribs. For sample H4 (deep beam), the average settlement depth was 1.35 mm below the rib lugs and 1.05 mm between the ribs. Similarly, for the wall element sample C10, the average settlement of concrete was 0.92 mm below the rebar ribs and 0.74 mm between the ribs. In the case of wall element sample L10, the air void under the rebar was caused by secondary plastic settlement that developed after the initial curing. Thus, in the direct vicinity of the rebar, the concrete cover bonded to the rebar, and due to the settlement, a crack formed that merged with trapped air pores, creating a discontinuity.

Larger air pores within the continuous void may also be identified beneath the top rebars. These pores were trapped below the surface of the rebar, and subsequently the crack created by settlement has connected them with the air void formed by the bleeding phenomenon.

These findings coincide with studies performed on panel elements made of self-compacting concrete [10]. The settlement depth in the cited research was smaller, ranging between 0.33 mm and 0.40 mm between ribs and between 0.63 mm and 1.18 mm under rib lugs. The panel elements used in the research were 480 mm high, whereas, in this study, the deep beam and wall element were 640 mm and 1600 mm high, respectively.

Irrespective of the position over height, there was a visible trend that a higher void volume was created under the bars than above them. However, this difference is significantly more pronounced for the rebars located in the upper part of the elements.

Furthermore, the research has demonstrated that, in the case of the top bars, regardless of the type of element and the distance from the casting location, a loss of structural continuity of the concrete occurs in the vicinity of the rebars. These bars, which are placed in a fixed position in the formwork, disrupt the plastic settlement of the mixture and cause shear-induced cracks to appear in their proximity (Figure 5 and Figure 6). The emergence of cracks results in an uneven stress distribution at the bar–concrete interface, a phenomenon that is not reflected in the current mathematical models that describe the bond phenomenon.

### 3.2. Effect of Distance from the Casting Point

The analysis of the porosity around the reinforcing bars indicates a tendency for the distance from the casting point to influence the amount of air voids in the SCI and the magnitude of settlement. However, this varies according to the size of the element.

In the case of the wall element, it is evident that there is an increase in the amount of air in the SCI of specimens located further away from the casting point than around it. This is evidenced by a slight increase in the porosity of the SCI of 3.06% for the lower specimens compared to 26.76% for the upper specimens. The size of voids beneath the top rebars associated with plastic settlement and bleeding increased by 48.20% for specimen L10 in comparison to specimen C10. The observed differences may be attributed to variations in the venting efficiency and the flow path of the concrete mixture. A study on bond [32] observed that the settlement of the concrete mixture in the formwork increases with distance from the point of concreting, which has a direct impact on the quality and size of the rebar cover.

In the case of the deep beam element, the same relationship between increased settlement under the bar and increased porosity of the SCI with increasing distance from the casting point was observed for the top specimens. This was, respectively, a 14.94% increase in the porosity of specimen H4 relative to B4 and a 15.89% increase in the void size under the rebar. Conversely, in the case of the bottom specimens, a higher-porosity SCI was observed in the specimen closer to the mix-casting point, specifically specimen B1. The increase in porosity observed in this specimen was associated with the appearance of several larger air pores within the SCI. However, these pores did not directly connect to the steel within the inter-rib space. The occurrence of larger voids below and above the individual ribs may also have been intensified by the inaccuracy of the tomographic measurements, which combined many small air bubbles into a single void. If these inaccuracies are disregarded, the SCI porosity of sample B1 is estimated to be 1.68%, a considerably lower value than that obtained from the unfiltered data (Table 3).

## 4. Effect of Element Size on Rebar–Concrete Interface and Its Implications for Standard Guidelines

Given the direct relationship between the quality of the reinforcing bar cover and its bond to the concrete, the observations regarding the effect of element size on the quality of SCI referred to the standard guidelines [3,4] for steel–concrete bond. In the currently valid European standard EN 1992 [4], the quality of the bond conditions varies according to the bar inclination relative to the horizontal and the position of the rebar relative to the top surface during concreting. In the case of rebars with an inclination of less than 45° to the horizontal, the zone of poor bond conditions is located up to 300 mm from the top surface of the element. In accordance with the American standard ACI 318-19 [3], the casting position factor remains dependent on the thickness of the fresh concrete layer beneath the horizontal reinforcement. For thicknesses exceeding 12 in (approximately 305 mm), bars are situated in zones of poor bond conditions. Consequently, one can conclude that the European standard assumes that bond conditions depend on the concrete layer above the bar, whereas the American standard assumes that bond conditions depend on the concrete layer below the bar. In the case of rebars with an inclination of less than 45° to the horizontal, the zone of poor bond conditions is located up to 300 mm from the top surface of the element. However, the bond conditions and, consequently, the design factors remain constant throughout the zone, regardless of the element height, which may be either 0.5 m or 5 m. Given that both elements are taller than 300 mm, the top bars are situated within the zone of poor bond conditions, as defined in both EN 1992 [4] and ACI 318 [3].

A review of the standard provisions indicates that the quality of the top rebars in the deep beam and wall element should be similar. This preliminary assumption is supported by the tomographic results, which indicate that the porosity of the zone around the top reinforcing bars of the deep beam element was approximately 6.98%, and that of the wall element was approximately 6.44%. Similarly, the size of the voids under the rebar is comparable in both cases, fluctuating around 2200 mm^3^. The depth of the air void resulting from the combined effects of plastic settlement and bleeding is also comparable in both instances. The SCI porosity-analysis images of the bottom specimens are also comparable in both elements and are characterized by significantly lower porosity compared to the top specimens. Furthermore, there was no evidence of plastic settlement under these bars. Therefore, it can be assumed that the element size will not affect the amount of settlement under the reinforcing bar and the porosity of the SCI for the two outermost bar positions in the element: the bottom bar and the bar close to the top surface of the element. It is notable that, in a tomographic study on a member made of ordinary concrete with a height of 1 m [11], it was observed that the amount of settlement under the rebar was related to the thickness of the concrete layer above the bar. As the thickness decreased, the size of the void under the bar also increased.

The extent of plastic settlement, which affects the quality of the concrete cover and its bond to the reinforcing bars, may vary depending on the dimensions of the element and the presence of confining elements such as formwork walls. The observations presented in this article regarding SCCs and in the work of Moccia et al. [11] regarding normal concretes are applicable to structural elements with narrow cross-sections (walls, beams, columns, etc.). In the case of slab or block elements, it is typical to observe increased plastic settlement in the central parts due to the lack of friction and confinement effect of the formwork walls. Additionally, it is noteworthy that this effect is overlooked in the standard guidelines.

## 5. Future Research Perspectives

These findings are promising, yet additional research is necessary to verify their accuracy beyond the tested range. For the future research directions, it appears to be important to verify SCI quality along the whole length and height of elements. An analysis of the influence of different rib patterns of rebars and the occurrence of corrosion on air-bubble trapping is suggested. It would also be valuable to investigate the possibility of improving the quality of the steel–concrete interface through modifications to the concrete composition, concreting technology, and location of the casting point. Based on the extended scope of research, it will be possible to understand how these changes will affect bond-test results and compliance with standards.

## 6. Conclusions

The effect of element size on the microstructure of the steel–concrete interface was studied using X-ray computed tomography image analysis on two types of elements: wall and deep beam. The key findings and conclusions are summarized below:

- Under the top rebars, the combined effects of bleeding and air-pore entrapment within the SCI were observed, which were subsequently connected by the crack induced by the plastic settlement of the concrete. The extent of these phenomena was independent of the height of the element.
- Comparable porosity rates of the SCC mix were observed under the top rebars in both the deep beam and wall elements, suggesting that the deterioration of the SCI is not related to the depth of the concrete layer under the rebar. Instead, it may be influenced by the distance from the top surface of the element.
- Larger settlement of the mixture was observed mainly under the rib lugs of the top bars (up to 2.69 mm wide), while smaller longitudinal voids were observed in the spaces between them (up to 1.66 mm wide). This trend may be caused by the shape of the ribs, which favors the slippage of the mix. The exceptions to this trend were air bubbles trapped between the ribs of the reinforcement, which were omitted in the average settlement estimation.
- In the vicinity of the bottom rebar, regardless of the element size, no significant SCI defects were observed. There was no concrete settlement, and most of the air voids were not directly connected to the steel surface.
- There was minor variation in the SCI quality of the rebars concerning their location relative to the casting point. This variation was more pronounced in the top part of the element, which may result from the flow path of the SCC mix and the tendency of the mix to drop, increasing with distance from the casting point.

## Figures and Tables

**Table 1:** Composition of SCC mix.

Ingredients	(kg/m^3^)
Blast-furnace cement (CEM III/A 42.5N)	450
Water	160
Sand (0–2 mm)	685
Gravel (2–8 mm)	510
Gravel (8–16 mm)	560
Fly ash	–
Superplasticizer	4.95
Plasticizer	2.25

**Table 2:** Fresh properties and compressive strength of the SCC.

Slump Flow (mm)	Slump Flow Time (s)	L-Box Ratio	Fresh Visual Stability Index	Compressive Strength (MPa)	Standard Deviation (MPa)
645	5.0	0.85	1	73.03	4.33

**Table 3:** Results of the porosity analysis in the vicinity of the rebar for void volumes ≥ 0.2 mm^3^.

Element	Sample	Row	Porosity (%)	Void Volume (mm^3^)		Void Number	
				Above Rebar	Below Rebar	Above Rebar	Below Rebar
Beam	B4	Top	6.49	150.61	2139.00	273	200
	B1	Bottom	3.37	385.30	805.26	487	449
	H4	Top	7.46	151.53	2478.93	181	170
	H1	Bottom	1.34	256.16	217.66	410	399
Wall	C10	Top	5.68	416.92	1586.93	393	341
	C1	Bottom	2.29	367.32	440.37	458	564
	L10	Top	7.20	188.79	2351.74	279	162
	L1	Bottom	2.36	343.85	489.87	425	539

## Data Availability

The raw data supporting the conclusions of this article will be made available by the authors upon request.

## References

[1] Angst U.M., Geiker M.R., Michel A., Gehlen C., Wong H., Isgor O.B., Elsener B., Hansson C.M., François R., Hornbostel K. (2017). The Steel–Concrete Interface. Mater. Struct..

[2] Powers T.C. (1968). The Properties of Fresh Concrete.

[3] (2019). Building Code Requirements for Structural Concrete.

[4] (2024). Design of Concrete Structures, Part 1-1: General Rules and Rules for Buildings, Bridges and Civil Engineering Structures.

[5] De Schutter G., Bartos P.J.M., Domone P. (2008). Self-Compacting Concrete.

[6] Zhang R., Castel A., François R. (2011). Influence of Steel–Concrete Interface Defects Owing to the Top-Bar Effect on the Chloride-Induced Corrosion of Reinforcement. Mag. Concr. Res..

[7] Soylev T.A., François R. (2003). Quality of Steel–Concrete Interface and Corrosion of Reinforcing Steel. Cem. Concr. Res..

[8] Azizinamini A., Stark M., Roller J.J., Ghosh S.K. (1993). Bond Performance of Reinforcing Bars Embedded in High-Strength Concrete. Struct. J..

[9] Castel A., Vidal T., Viriyametanont K., François R. (2006). Effect of Reinforcing Bar Orientation and Location on Bond with Self-Consolidating Concrete. ACI Struct. J..

[10] Dybeł P. (2021). Effect of Bottom-up Placing of Self-Compacting Concrete on Microstructure of Rebar-Concrete Interface. Constr. Build. Mater..

[11] Moccia F., Kubski X., Fernández Ruiz M., Muttoni A. (2021). The Influence of Casting Position and Disturbance Induced by Reinforcement on the Structural Concrete Strength. Struct. Concr..

[12] Withers P.J., Bouman C., Carmignato S., Cnudde V., Grimaldi D., Hagen C.K., Maire E., Manley M., Du Plessis A., Stock S.R. (2021). X-ray Computed Tomography. Nat. Rev. Methods Primers.

[13] Kaczmarczyk G.P., Cała M. (2023). Possible Application of Computed Tomography for Numerical Simulation of the Damage Mechanism of Cementitious Materials—A Method Review. Buildings.

[14] Zehong W., Ya W., Shuangjie W., Jianbing C. (2020). Application of X-ray Micro-CT for Quantifying Degree of Hydration of Slag-Blended Cement Paste. J. Mater. Civ. Eng..

[15] Kim H.T., Park K. (2022). Computed Tomography (CT) Image-Based Analysis of Concrete Microstructure Using Virtual Element Method. Compos. Struct..

[16] Maaroufi M., Abahri K., El Hachem C., Belarbi R. (2018). Characterization of EPS Lightweight Concrete Microstructure by X-ray Tomography with Consideration of Thermal Variations. Constr. Build. Mater..

[17] Nguyen T., Ghazlan A., Kashani A., Bordas S., Ngo T. (2018). 3D Meso-Scale Modelling of Foamed Concrete Based on X-ray Computed Tomography. Constr. Build. Mater..

[18] Ponikiewski T., Gołaszewski J., Rudzki M., Bugdol M. (2015). Determination of Steel Fibres Distribution in Self-Compacting Concrete Beams Using X-ray Computed Tomography. Arch. Civ. Mech. Eng..

[19] Ahmed H., Kuva J., Punkki J. (2024). Analysing Entrapped Pores in Concrete via X-ray Computed Tomography: Influence of Workability and Compaction Time. Constr. Build. Mater..

[20] Sadowski Ł., Stefaniuk D. (2018). The Effect of Surface Treatment on the Microstructure of the Skin of Concrete. Appl. Surf. Sci..

[21] Yuan J., Wu Y., Zhang J. (2018). Characterization of Air Voids and Frost Resistance of Concrete Based on Industrial Computerized Tomographical Technology. Constr. Build. Mater..

[22] Taheri-Shakib J., Al-Mayah A. (2024). Effect of Corrosion Pit Distribution of Rebar on Pore, and Crack Characteristics in Concrete. Cem. Concr. Compos..

[23] Björngrim N., Myronycheva O., Fjellström P.A. (2022). The Use of Large-Scale X-ray Computed Tomography for the Evaluation of Damaged Structural Elements from an Old Timber Bridge. Wood Mater. Sci. Eng..

[24] Jaskowska-Lemańska J., Wałach D., Górka-Stańczyk M. (2023). Correction Factors for Sclerometric Test Results in the Technical Assessment of Timber Structural Elements under Diverse Conditions. Materials.

[25] Ziółkowski G., Chlebus E., Szymczyk P., Kurzac J. (2014). Application of X-ray CT Method for Discontinuity and Porosity Detection in 316L Stainless Steel Parts Produced with SLM Technology. Arch. Civ. Mech. Eng..

[26] Fan J., Li Y., Gao Y., Zhang X., Jiang P. (2021). Evaluation of the Morphology and Pore Characteristics of Silica Refractory Using X-ray Computed Tomography. Ceram. Int..

[27] (2012). Cement—Part 1: Composition, Specifications and Conformity Criteria for Common Cements.

[28] (2010). Testing Fresh Concrete, Part 8: Self-Compacting Concrete—Slump-Flow Test.

[29] (2010). Testing Fresh Concrete, Part 10: Self-Compacting Concrete—L-Box Test.

[30] (2018). Standard Test Method for Slump Flow of Self-Consolidating Concrete.

[31] (2007). Steel for the Reinforcement of Concrete.

[32] Thrane L.N., Pade C., Idzerda C., Kaasgaard M. (2010). Effect of Rheology of SCC on Bond Strength of Ribbed Reinforcement Bars. Design, Production and Placement of Self-Consolidating Concrete.

